# Selectivity and Reactivity of Zr^IV^ and Ce^IV^ Substituted Keggin Type Polyoxometalates Toward Cytochrome c in Surfactant Solutions

**DOI:** 10.3389/fchem.2018.00372

**Published:** 2018-08-28

**Authors:** Thomas Quanten, Tessa De Mayaer, Pavletta Shestakova, Tatjana N. Parac-Vogt

**Affiliations:** ^1^Laboratory of Bio-Inorganic Chemistry, Department of Chemistry, KU Leuven, Leuven, Belgium; ^2^NMR Centre, Institute of Organic Chemistry with Centre of Phytochemistry, Bulgarian Academy of Sciences, Sofia, Bulgaria

**Keywords:** polyoxometalates, Keggin, protein, hydrolysis, surfactants, cytochrome c

## Abstract

In this paper we investigate the effect of three different types of surfactants, on the hydrolysis of Cytochrome c (Cyt c), a predominantly α helical protein containing a heme group, promoted by [Ce(α PW_11_*O*_39_)2]10- (CeK) and [Zr(α PW_11_*O*_39_)2]10- (ZrK) polyoxometalates. In the presence of SDS, Zw3 12, or CHAPS surfactants, which are commonly used for solubilizing hydrophobic proteins, the specificity of CeK or ZrK toward hydrolysis of Cyt c does not change. However, the hydrolysis rate of Cyt c by CeK was increased in the presence of SDS, but decreased in the presence of CHAPS, and was nearly inhibited in the presence of Zw3 12. The Circular dichroism and Tryptophan fluorescence spectroscopy have shown that the structural changes in Cyt c caused by surfactants are similar to those caused by POMs, hence the same specificity in the absence or presence of surfactants was observed. The results also indicate that for Cyt c hydrolysis to occur, large unfolding of the protein is needed in order to accommodate the POMs. While SDS readily unfolds Cyt c, the protein remains largely folded in the presence of CHAPS and Zw3 12. Addition of POMs to Cyt c solutions in CHAPS results in unfolding of the structure allowing the interaction with POMs to occur and results in protein hydrolysis. Zw3 12, however, locks Cyt c in a conformation that resists unfolding upon addition of POM, and therefore results in nearly complete inhibition of protein hydrolysis.

## Introduction

Membrane proteins perform key functions in crucial processes at the interface between the intra- and extracellular environment (Tan et al., 2008). Atypical or dysfunctional behavior of membrane proteins often presents itself in a range of diseases. Identifying these abnormal membrane proteins might result in novel therapeutic targets (Carter et al., 2004). Approximately 60% of known and future drug targets are membrane proteins (Hopkins and Groom, 2002; Overington et al., 2006). Unfortunately, membrane proteins are often underrepresented in proteomic studies (Santoni et al., 2000; Whitelegge et al., 2003; Seddon et al., 2004; Speers and Wu, 2007; Tan et al., 2008; Rabilloud, 2009), even though the predicted number of membrane associated proteins is sizable (Wallin and von Heijne, 1998). Their limited representation is attributed to their low abundance and their heterogeneous and hydrophobic nature. To solubilize membrane proteins in aqueous solutions, surfactants are often employed. The surfactants form micelles or liposomes that mimic the hydrophobic environment of the cell membrane. Unfortunately, proteolytic enzymes are mostly hydrophilic and tend to denature in the presence of surfactants, resulting in the loss of catalytic activity. Therefore, artificial peptidases that remain active in the presence of surfactants need to be developed.

Proteomics is a field of science that studies the proteome, i.e., the entire population of proteins of a cell, tissue or organism, mostly by means of mass spectrometry (MS) techniques. Intact proteins are often too large to study via MS and are therefore digested in a controlled fashion. Digestion of proteins is achieved by controlled hydrolysis of the peptide bonds that link amino acids in a protein. However, the half-life of peptide bonds under physiological conditions has been estimated to be between 350 and 600 years (Bryant and Hansen, 1996; Radzicka and Wolfenden, 1996; Smith and Hansen, 1998). To accelerate protein digestions, hydrolytic enzymes are employed. A well-known example is trypsin which specifically targets the *C*-terminal peptide bond of lysine (Lys) and arginine (Arg) residues (Rodriguez et al., 2008). However, the frequency with which these residues occur results in many fragments that are too small (56% of fragments contain ≤ 6 residues) to be confidentially detected and analyzed by MS techniques (Swaney et al., 2010; Tsiatsiani and Heck, 2015). Additionally, most of the currently available hydrolytic enzymes produce fragments between 0.5 and 3 kDa in size. Such short fragments are not compatible with the emerging field of middle-down proteomics that foccuses on the analysis of middle-sized fragments which are typically 10–15 kDa.

Most of the chemical agents used for peptide bond hydrolysis reported so far require harsh conditions to achieve efficient cleavage of the targeted peptide bonds. These conditions often chemically modify some amino acids and the native terminal groups. Transition metals and their complexes hold great potential as synthetic enzymes. Numerous examples of metal ion promoted peptide bond hydrolysis in peptides, oligopeptides and proteins in aqueous solutions have been reported (Grant and Kassai, 2006; Wezynfeld et al., 2016). However, the selective hydrolysis of proteins in the presence of surfactants has been largely unexplored and only two examples involving Pt^II^ and Pd^II^ complexes have been reported albeit at very acidic pH conditions (2.5–2.9) (Milovic et al., 2003; Miskevich et al., 2011).

Polyoxometalates or POMs are anionic, oligomeric aggregates of early transition metal ions bridged by oxo ligands with versatile physical and chemical properties (Pope, 1983; Pope and Müller, 1991; Hasenknopf, 2005). Due to their versatile nature POMs are employed in numerous fields such as catalysis, material science and medicine (Rhule et al., 1998; Sadakane and Steckhan, 1998; Kozhevnikov, 2002; Long et al., 2007, 2010; Wang and Yang, 2015). Investigating their activity toward biomolecules and model systems has demonstrated their unprecedented carboxyesterase, phosphoesterase, peptidase, and sialidase activity (Hegg and Burstyn, 1998; Absillis et al., 2008, 2011; Cartuyvels et al., 2008; Lokeren et al., 2008; Steens et al., 2009, 2010; Ho et al., 2011a,b, 2012; Absillis and Parac-Vogt, 2012; Vanhaecht et al., 2012, 2013; Ly et al., 2013a,b, 2015b; Luong et al., 2014, 2015a,b, 2016, 2017; Stroobants et al., 2014c; Van Rompuy and Parac-Vogt, 2017).

Ce^IV^ and Zr^IV^ substituted POMs were shown to promote selective hydrolysis of a variety of water soluble proteins, ranging from flexible polypeptide such as oxidized insulin chain B (Sap et al., 2015), to larger, globular proteins such as hen egg white lysozyme (Stroobants et al., 2013, 2014d), human serum albumin (HSA) (Goovaerts et al., 2013; Stroobants et al., 2014a,b), horse heart cytochrome c (Cyt c) (Sap et al., 2016) and horse heart myoglobin (Ly et al., 2015a; Ly and Parac-Vogt, 2017). However, the potential of these POMs to act as artificial proteases toward hydrolysis of proteins in the presence of surfactants is still largely unexplored. We have previously investigated the interaction between metal-substituted POMs and different surfactants with the help of ^31^P and ^1^H DOSY NMR spectroscopy and demonstrated that the POM retains its catalytic activity in the presence of surfactants (Quanten et al., 2016). Sap et al. later investigated the effect of the zwitterionic surfactant 3-[(3-Cholamidopropyl)dimethylammonio]-1- propanesulfonate, also known as CHAPS, on the activity and selectivity of [Zr(α_2_-P_2_W_17_O_61_)_2_]^16−^ toward the hydrolysis of the HSA (Sap et al., 2017). In the absence of surfactants, HSA was cleaved at four sites (Arg114-Leu115, Ala257-Asp258, Lys313-Asp314, and Cys302-Glu303), while hydrolysis of seven peptide bonds (Cys62-Asp63, Gly71-Asp72, Asp107-Asp108, Lys313-Asp314, His367-Gly368, Ser470-Asp471, and Ala511-Asp512) was observed in the presence of 0.5% CHAPS. Interestingly, most of the POM-targeted peptide bonds were at an aspartic acid or glutamic acid residue, and the POM affinity to cleave at these amide bonds remained the same in the presence of 0.5% CHAPS.

**Figure 1:**
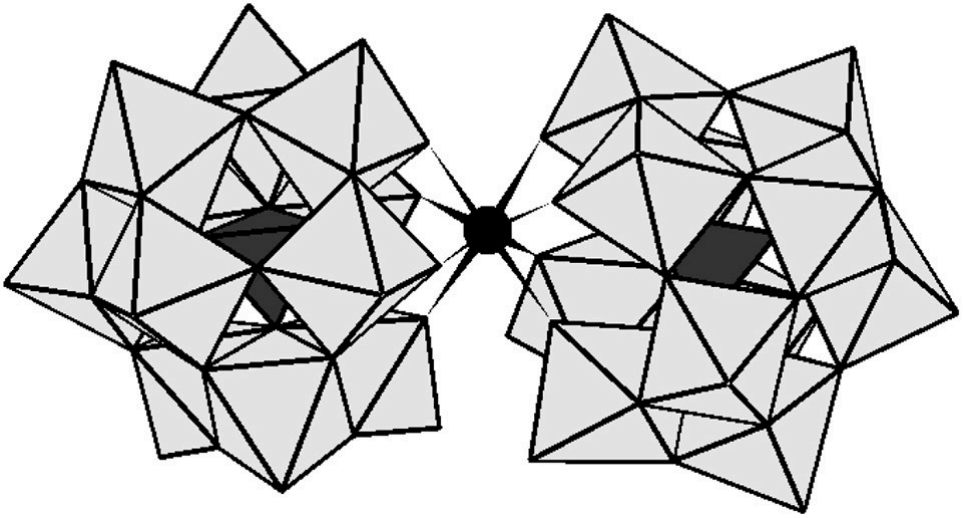
The polyhedral representation of [Ce(α-PW_11_O_39_)_2_]^10−^ and [Zr(α-PW_11_O_39_)_2_]^10−^. The gray octahedral represents WO_6_ and the black tetrahedral stand for PO_4_. The black sphere represents Zr^IV^ or Ce^IV^.

**Figure 2:**
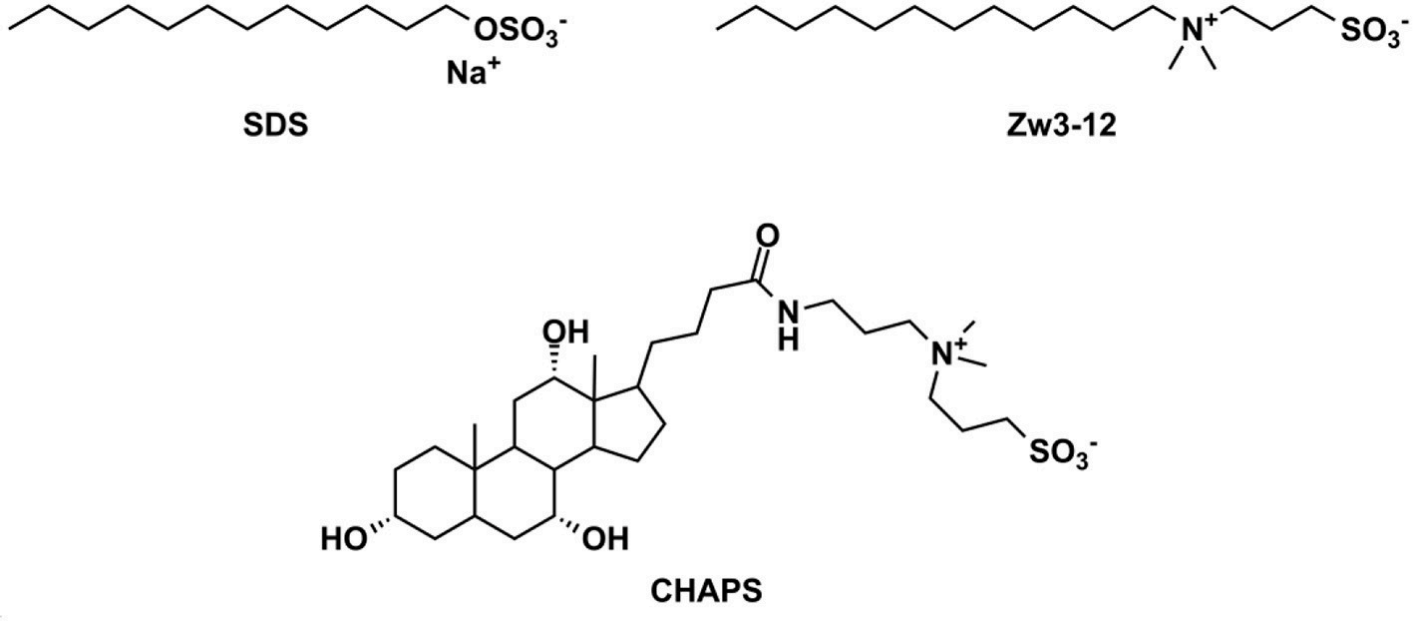
The chemical structures of the surfactants used in this study. SDS, sodium dodecyl sulfate; Zw3-12, dodecyldimethyl(3-sulfopropyl)ammonium and CHAPS; 3-[(3-cholamidopropyl)dimethylammonio]-1-propanesulfonate.

Encouraged by this result, in this paper we investigate the effect of three different types of surfactants on the hydrolysis of Cytochrome c (Cyt c), a predominantly α-helical protein containing a heme group (Bushnell et al., 1990), promoted by [Ce(α-PW_11_O_39_)_2_]^10−^ (**CeK**) and [Zr(α-PW_11_O_39_)_2_]^10−^ (**ZrK**) polyoxometalates. In comparison to HSA, Cyt c is a small globular protein consisting out of 104 amino acids with a net positive charge of +8 at neutral pH and an isoelectric point of almost 10. Due to its smaller size and a net positive charge, the interaction of Cyt c by POM catalysts is likely to be affected by the presence of different surfactants. In order to elucidate the role of surfactants on the catalytic performance of POMs, in this work we study the hydrolysis of Cyt c by **CeK** and **ZrK** (see Figure 1) in the presence of surfactants shown in Figure 2. Furthermore, a range of spectroscopic techniques was used to gain insight into the molecular interactions between **CeK** and **ZrK** POMs and Cyt c in different surfactant solutions.

## Results and discussion

### Hydrolysis of Cyt c by CeK and ZrK in the presence of different surfactants

**Figure 3:**
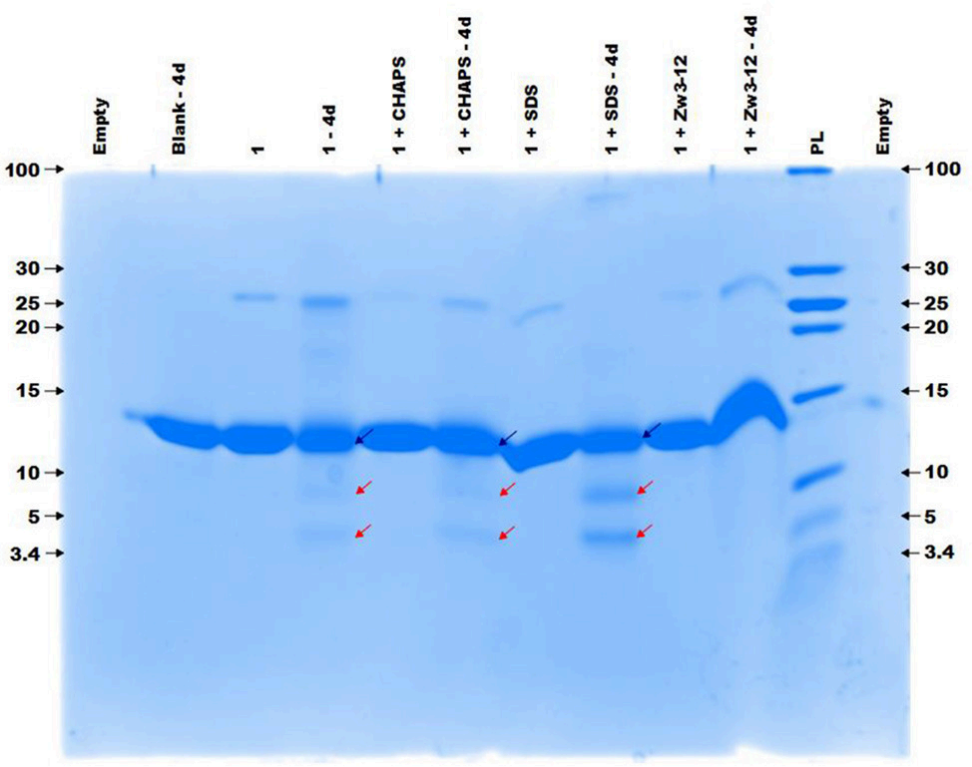
The colloidal Coomassie blue (CCB) stained gel of the hydrolysis of 40 μM Cyt c by 2 mM of 1 in the absence or presence of different surfactants. Cyt c was incubated for 4 days at 60°C and pH 7.4 (10 mM sodium phosphate buffer). The blue and red arrows indicate the intact protein and the generated fragments at 7.8 and 3.9 kDa, respectively. The values at the left and right side of the gel indicate the Mw reference in kDa. An aliquot of each sample was taken after mixing and 4 days (4d) of incubation, except for the blank (Cyt c without 1 or surfactants present) which only shows an aliquot after 4 days. The content of each lane is shown on top of the gel and the concentration of each surfactant was kept at 0.5%. PL stand for protein ladder.

Previous research has shown that **CeK** and **ZrK** specifically cleaved Cyt c in aqueous solution, but at different sequences. While **CeK** cleaved Cyt c at Trp60-Lys61 and Gly78-Thr79 peptide bonds at pH 7.4, the **ZrK** splits three peptide bonds: Asp3-Val4, Asp51-Ala52, and Gly78-Thr79 at pH 4.1 (Sap et al., 2016). These differences in selectivity are likely due to the oxido-reduction processes that occur during protein hydrolysis by CeK, as evidenced by ^31^P NMR spectroscopy, but are absent during Cyt c hydrolysis by Zr POMs. These oxido-reduction processes can change the folding of Cyt c through a modification of the side-chains of redox-active amino acids and can also change the coordination ability of Ce due to a change in its oxidation state (Ce^IV^ to Ce^III^). In this work we examine the effect of three different surfactants, which are used in the study of membrane proteins, on the selectivity and the efficiency of the hydrolysis. These surfactants differ in their charge and polarity and are therefore expected to exhibit different interactions with POMs and proteins. Zw3-12 and SDS have the same hydrophobic dodecyl chain, however SDS is anionic while Zw3-12 is zwitterionic in nature. CHAPS has the same zwitterionic head group as Zw3-12, but as a tail it has hydroxyl functionalized steroid skeleton, making it the most hydrophilic surfactant among the examined three (see Figure 2). As can be seen in Figure 3, in the absence of surfactants **CeK** cleaves Cyt c in two fragments with a molecular weight (Mw) of 7.8 and 3.9 kDa, which are the same as previously observed by Sap et al. When 0.5% CHAPS or 0.5% SDS were added to the incubation mixture, the same fragments were observed, indicating that these surfactants do not alter the specificity of **CeK**. Interestingly, addition of 0.5% Zw3-12, resulted in complete inhibition of the catalytic activity of **CeK** as no hydrolysis of Cyt c was observed in the SDS-PAGE.

**Figure 4:**
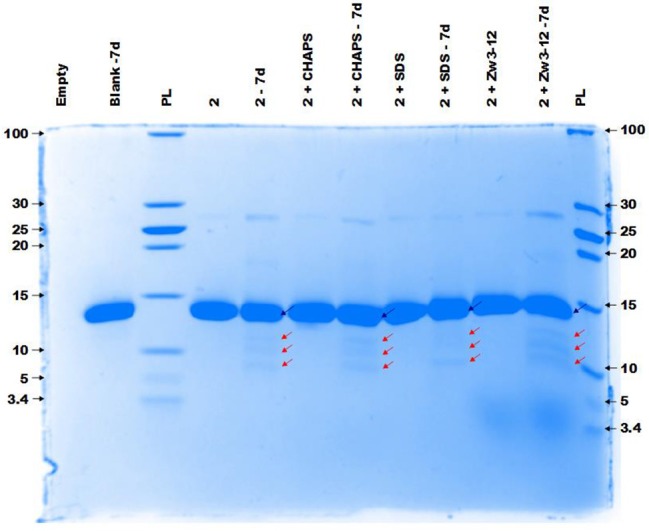
The CCB stained gel of the hydrolysis of 40 μM Cyt c by 2 mM of 2 in the presence or absence of different surfactants. Cyt c was incubated for 7 days at 60°C and pH 7.4 (10 mM sodium phosphate buffer). The blue and red arrows indicate the intact protein and the generated fragments at 10.3, 7.6, and 5.6 kDa, respectively. The values at the left and right side of the gel indicate the Mw reference in kDa. An aliquot of each sample was taken after mixing and 7 days (7d) of incubation, except for the blank (Cyt c without 1 or surfactants present) which only shows an aliquot after 7 days (7d). The content of each lane is shown on top of the gel and the concentration of each surfactant was kept at 0.5%. PL stand for protein ladder.

Figure 4 demonstrates the selective cleavage of Cyt c in the presence of **ZrK**, which results in three fragments with Mw 10.3, 7.6 and 5.6 kDa at pH 7.4. These fragments have the same Mw as those previously observed at pH 4.1 (10 mM acetate buffer) by Sap *et al*. Interestingly, the addition of CHAPS, SDS or Zw3-12 surfactants does not have a significant effect on the specificity of **ZrK**, as the same fragments were observed in the absence or presence of these surfactants.

Comparison of the results shown in Figures 3, 4 reveals that the most striking difference between **CeK** and **ZrK** is their efficiency as catalyst for the hydrolysis of Cyt c. Where **CeK** was able to selectively hydrolyze most of intact Cyt c after 4 days, hydrolysis of Cyt c by **ZrK** was only noticeable after 7 days of incubation at 60°C. This difference is most likely the result of the different Lewis acidic strength of both metals. Cerium is characterized by a stable 3+ and 4+ oxidation state, while zirconium is only stable in a 4+ state. This difference allows Ce^IV^ to take up more electron density than Zr^IV^, resulting in a stronger Lewis acidity for Ce^IV^. Another interesting observation is that the rates of protein hydrolysis were differently influenced by three surfactants. The rate of protein hydrolysis could be assessed by measuring the intensity decrease of the intact protein band upon incubation at 60°C. Table 1 summarizes the amount of hydrolyzed Cyt c measured in the absence or presence of different surfactants.

**Table 1:** The breakdown of Cyt c by CeK and ZrK after incubation at 60°C and pH 7.4 (10 mM sodium phosphate buffer).

**Surfactant (0.5%)**	**Cyt c breakdown by CeK (%)^a^**	**Cyt c breakdown by ZrK (%)^b^**
–	24	18
SDS	44	4
CHAPS	21	17
Zw3-12	–	9

a *after 4 days of incubation*.

b *after 7 days of incubation*.

Interestingly, in 0.5% SDS solutions of **CeK**, the yield of protein hydrolysis by **CeK** nearly doubled. While only 24% of Cyt c was cleaved in the absence of any surfactants, 44% hydrolysis of Cyt c was observed after 4 days in the presence of 0.5% SDS. In the presence of 0.5% CHAPS 21% of Cyt c was fragmented, which is similar to the hydrolysis rate in the absence of surfactants. In the presence of 0.5% Zw3-12, however, no degradation of Cyt c was observed after 4 days.

Compared to **CeK**, **ZrK** is an overall less efficient catalyst. At pH 7.4. Sap et al. also observed weak hydrolysis at this pH, but were able to observe more efficient hydrolysis of Cyt c in more acidic solutions (pH 4.1) (Sap et al., 2016). At pH 7.4, **ZrK** was able to degrade 18% of Cyt c in absence of surfactants. CHAPS had very little effect on the hydrolysis yield, but adding 0.5% of SDS or Zw3-12, slightly lowered the Cyt c digestion to 4 and 9%, respectively.

These findings suggest that the nature of the surfactant plays a role in the hydrolytic activity of **CeK** and **ZrK** toward Cyt c. Therefore the molecular interactions between Cyt c and **CeK** and **ZrK** in the absence or presence of the different surfactants were studied with the help of different spectroscopic techniques in order to elucidate the origin of the observed specificity and reactivity of **CeK** and **ZrK** in the absence or presence of different surfactants.

### Effect of CeK, ZrK and surfactants on the secondary and tertiary structure of Cyt c

The structural changes caused in Cyt c by **CeK**, **ZrK** and/or surfactants were studied with circular dichroism (CD) spectroscopy. The conformation of the protein backbone is reflected in the far-UV region of a CD spectrum, making it a useful technique to study the secondary structure of a protein. The far-UV CD spectra of Cyt c recorded in the absence or presence of 0.5% SDS or 0.5% Zw3-12 are shown in Figure S1 (see Supporting Information). Due to the strong absorption of CHAPS in the far-UV region, it was not possible to use it in the far-UV CD measurements.

**Figure 5:**
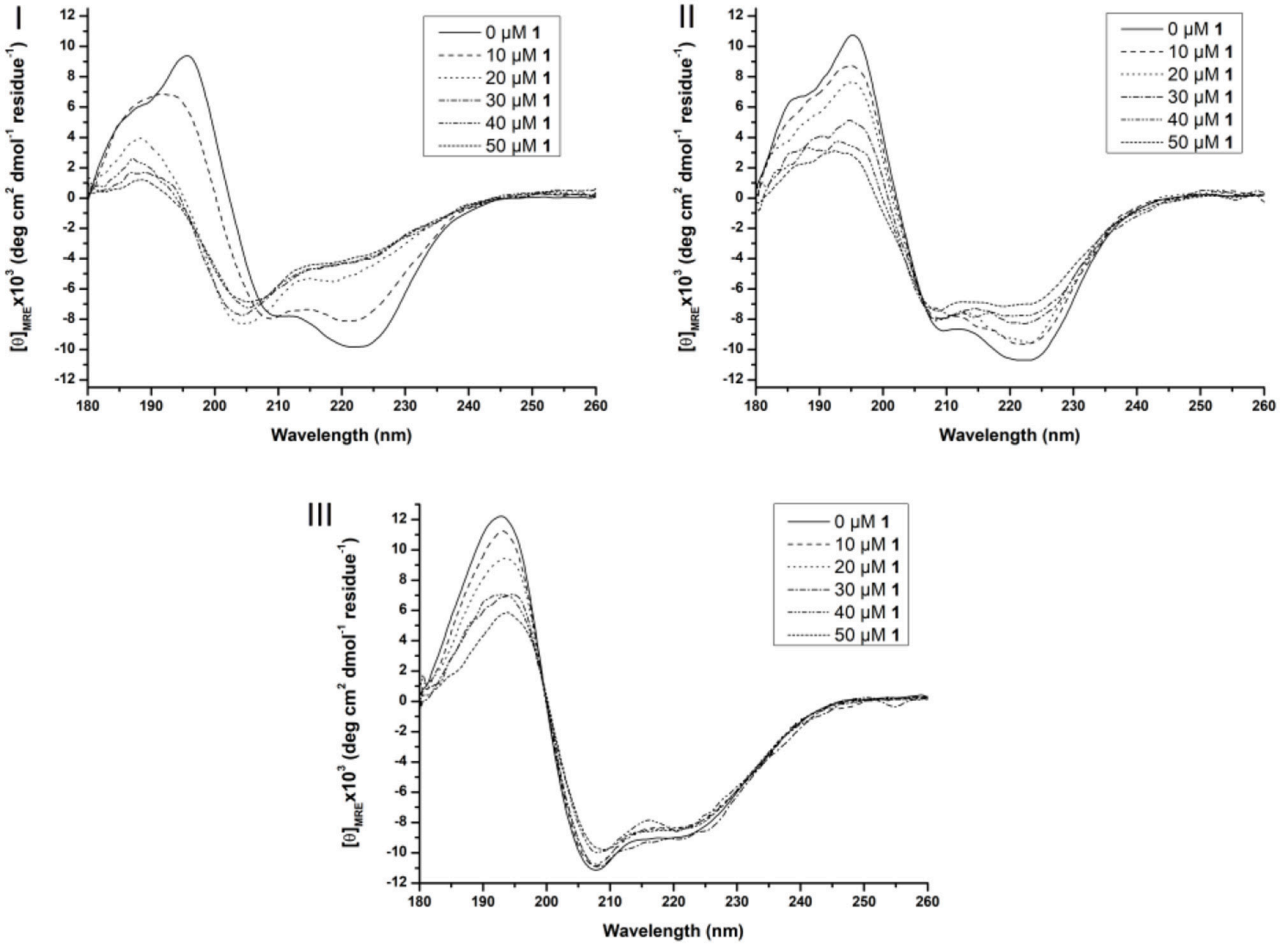
Far-UV CD spectra of 10 μM Cyt c solutions in presence of increasing concentration of 1 (from 0 to 50 μM) in the absence of surfactants **(I)**, in the presence of 0.5% Zw3-12 **(II**) and 0.5% SDS **(III)**. All samples were buffered at pH 7.4 by a 10 mM sodium phosphate buffer and kept at 25 ± 0.1°C during measurements.

As can be seen from Figure S1 SDS and Zw3-12 have a different influence on the secondary structure of Cyt c. The zwitterionic surfactant Zw3-12 does not change the overall shape of the CD spectrum but increases the intensity of the negative signals at 222 and 210 nm and positive signals at 195 and 185 nm. SDS, however, causes a larger change of the shape of the CD signal, as can be seen by the emergence of a strong negative signal at 208 nm and strong positive signal at 193 nm. Hiramatsu et al. have observed a similar effect of SDS on the secondary structure of Cyt c, where the negative signals at 209 and 222 nm disappeared in favor for a new negative trough at 207 nm (Hiramatsu and Yang, 1983). These observations were rationalized by an altered helicity of Cyt c in the presence of SDS, however the observed structural changes were not quantified. In this work DICHROWEB was employed to estimate the secondary structural content of Cyt c in the absence or presence of different surfactants and/or POMs. The far-UV region of a CD spectrum is the sum of the CD signals from the individual structural components (Sreerama and Woody, 2000; Whitmore and Wallace, 2004, 2008). DICHROWEB exploits this cumulative nature to deconvolute the various contributions of the different secondary structure elements (α-helices, β-sheets and random coils) with the help of protein reference sets and empirical algorithms (Whitmore and Wallace, 2004, 2008; Greenfield, 2006). DICHROWEB could provide calculated secondary structure contents of Cyt c in the presence or absence of surfactants, which are summarized in Table S1 (see Supporting Information). The calculated secondary structure content of Cyt c in the absence of surfactants is similar to that previously reported in literature (see first entry Table S1, Supporting Information) (Nantes et al., 2001). The calculated content confirms that Zw3-12 has a minor influence on protein structure, increasing the α-helical content by 3% and lowering the β-sheet content by the same amount, while SDS causes a restructuring of Cyt c by enhancing both the α-helical and β-sheet content by 2 and 6%, respectively. Next, the effect of **CeK** and **ZrK** on the structure of Cyt c was investigated in the absence or presence of Zw3-12 or SDS (see Figure 5 and Figure S2, respectively).

In solution, Cyt c assumes a predominantly α-helical structure as demonstrated by the negative signals at 209 and 222 nm and positive signal at 195 nm. Adding **CeK** to Cyt c in the absence of surfactants decreases the intensity of the signals at 209 nm and 222 nm and a new negative signal emerges at 205 nm. Additionally, the two positive signals at 186 and 195 nm disappear in favor for a new positive peak at 189 nm (see Figure 5I). After analyzing these CD spectra, it was calculated that the native Cyt c in solution contains 32 and 12% of α-helix and β-sheets, respectively (see Table S2). After adding 50 μM of **CeK** to Cyt c the secondary structure contents changed to 8% α-helix (24% decrease) and 34% β-sheets (23% increase). In the presence of 0.5% SDS or 0.5% Zw3-12 the structural changes are less pronounced (see Figures 5II, III). The structure that Cyt c assumes in the presence of Zw3-12, but in the absence of POMs, is similar to its structure in the absence of surfactants and POMs. However, when the concentration of **CeK** is increased, the structural changes are limited, as seen in the gradual decrease of signal intensity in Figure 5II. Adding 50 μM of **CeK** to a Zw3-12/Cyt c solution (0.5% Zw3-12) caused a 16% decrease in α-helix and 19% increase in β-sheet content (see Table S2). In the presence of 0.5% SDS, the restructuring effect of **CeK** is even smaller as seen in Figure 5III. The calculated structural content is summarized in Table S2 where it can be seen that change in secondary structure content in the absence or presence of **CeK** is rather limited (≤4%). These observations can be explained by competition between **CeK** and the surfactant for binding sites at Cyt c. This competitive model lowers the binding strength between **CeK** and Cyt c, explaining the limited influence of **CeK** on the secondary structure in the presence of surfactants. The strong anionic nature of SDS repels the polyanionic POM stronger than the zwitterionic Zw3-12 resulting in a lower binding strength in the presence of SDS compared to Zw3-12. These observations agree with a competitive model. An alternative explanation involves the surfactant locking Cyt c in a structure, which is only marginally influenced by the binding of **CeK**. Similar observations were made for the Zr^IV^ substituted POM, **ZrK** (see Figure S2 and Table S2), where the strongest influence of **ZrK** on the secondary structure is seen in absence of surfactants and this influence decreases when shifting from Zw3-12 to SDS. This indicates that the POM skeleton, and not the imbedded metal ion, plays the most significant role in altering the structure of Cyt c.

**Figure 6:**
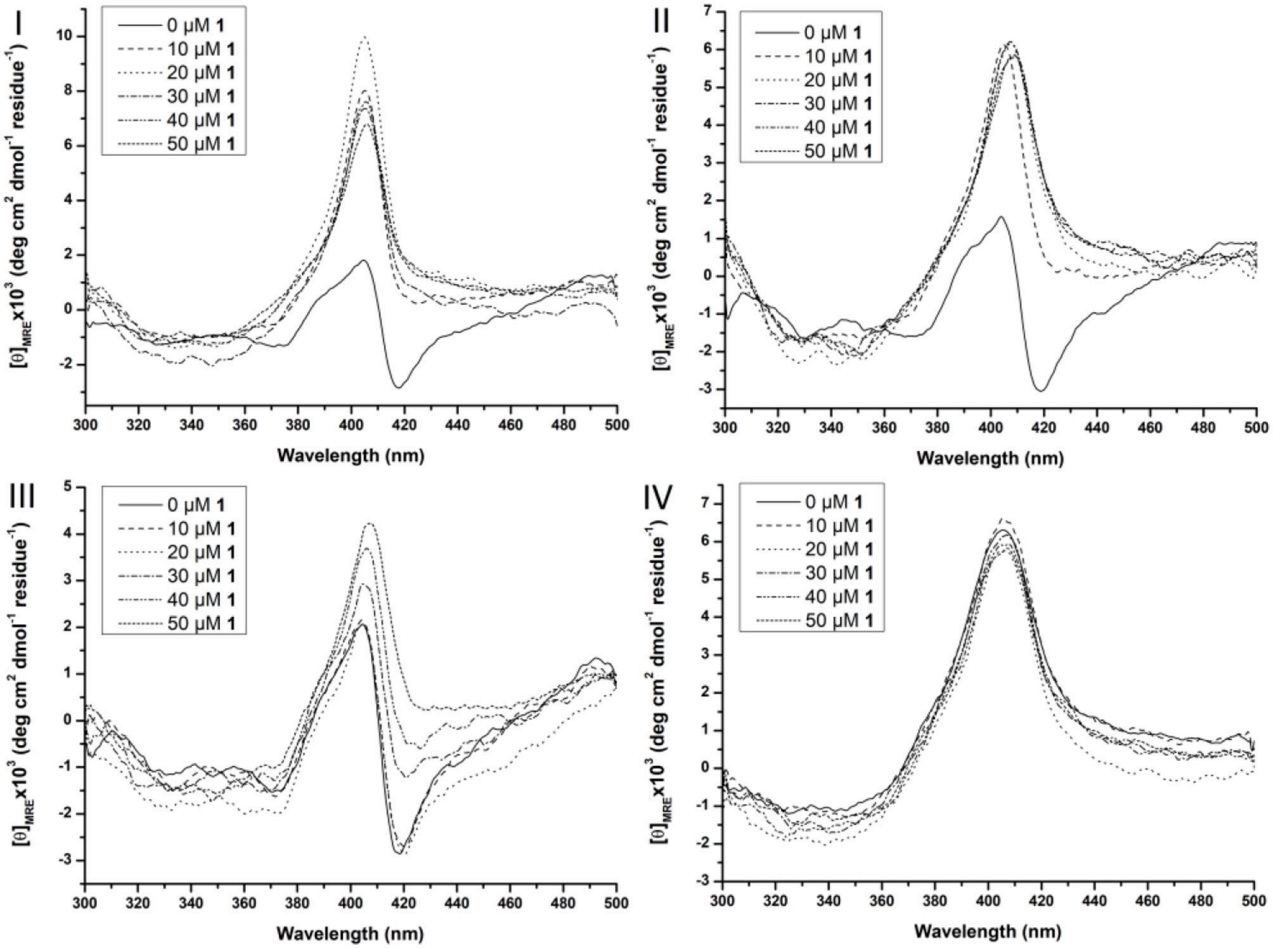
Soret region CD spectra of 10 μM Cyt c solutions in the presence of increasing concentration of CeK (1) (from 0 to 50 μM) in the absence of surfactants **(I)**, in the presence of 0.5% CHAPS **(II)**, 0.5% Zw3-12 **(III)**, and 0.5% SDS **(IV)**. All samples were buffered at pH 7.4 by a 10 mM sodium phosphate buffer and kept at 25 ± 0.1°C during measurements.

At higher wavelengths (300–500 nm) the CD spectrum of Cyt c is characterized by a Soretbisignate signal with a negative and positive peak at 418 and 404 nm, respectively. This bisignate band is assigned to the heme-polypeptide interactions close to the heme crevice and indicative for a natively folded Cyt c (Myer, 1968a,b; Kaminsky et al., 1972; Santucci and Ascoli, 1997; Thomas et al., 2000; Mugnol et al., 2008; Wei and Danielson, 2011). When Cyt c unfolds the negative signal disappears, while the positive signal becomes more intense (Santucci and Ascoli, 1997; Thomas et al., 2000; Mugnol et al., 2008; Wei and Danielson, 2011). The effect of the surfactants on the tertiary structure can be seen in Figure S4. Zw3-12 and CHAPS do not denature Cyt c as evidenced by the unaltered bisignate band around 410 nm in the presence of 1.0% CHAPS or Zw3-12. SDS, however, causes the negative signal at 418 nm to disappear, leaving only the positive signal at 406 nm. This suggests that Cyt c is largely denatured in the presence of 0.25% SDS as a result of its harsh nature. The effect of **CeK** and **ZrK** on the tertiary structure of Cyt c was also determined in the absence or presence of different surfactants (see Figure 6 and Figure S3, respectively).

In the absence of surfactants, large unfolding of Cyt c is observed in the presence of 10 μM **CeK**, indicated by the disappearance of the negative signal at 418 nm and appearance of a positive signal at 406 nm. A similar trend was observed in the presence of 0.5% CHAPS. In the presence of 0.5% Zw3-12, however, increasing the concentration of **CeK** caused a more gradual unfolding of Cyt c. The signal at 418 nm gradually disappeared when the amount of **CeK** increased and was completely suppressed at 50 μM. Simultaneously, the positive signal around 406 nm increased. The observed differences between Zw3-12 and CHAPS can be explained by their hydrophobic moiety. Zw3-12 has a saturated, linear dodecyl chain, while CHAPS has a polar, functionalized steroid skeleton. This makes Zw3-12 a more hydrophobic surfactant, resulting in a stronger interaction with Cyt c, and making it more difficult to be displaced by **CeK**. As a result, **CeK** has a weaker denaturing effect on Cyt c in the presence of Zw3-12 than in the presence of CHAPS. The final surfactant, SDS is known for its harsh denaturing effect on globular proteins. At 0.25%, SDS completely unfolds Cyt c, as evidenced by the loss of the negative part of the bisignateSoret band around 410 nm. The unfolded state of Cyt c in the presence of 0.5% SDS is comparable to its unfolded state in the presence of 50 μM **CeK** (compare Figure 6I with Figure 6IV). This might explain why adding 50 μM of **CeK** to a SDS:Cyt c mixture causes no changes in tertiary structure. The same trends are observed for **ZrK** (see Figure S3), proving again that the POM framework is the main determinant in the refolding of Cyt c in the presence of metal substituted Keggin POMs.

The hydrolysis rate of Cyt c by **CeK** is the fastest in the presence of 0.5% SDS, while in the presence of 0.5% CHAPS and in absence of surfactants the rates are comparable. In the presence of 0.5% Zw3-12, however, the hydrolysis is strongly inhibited. The reaction rate appears to be related to the unfolded state of Cyt c under these conditions. In the presence of SDS, Cyt c is largely unfolded and this new conformation closely resembles the structure that Cyt c assumes when binding with **CeK** or **ZrK**. In the absence of surfactants or in the presence of CHAPS these conformations are very similar, and increased POM concentrations result in large unfolding of Cyt c. Zw3-12, however, appears to lock Cyt c in a folded conformation that is rather resistant to unfolding upon addition of POMs. This resistance to denaturing might explain the inhibiting effect of Zw3-12 on Cyt c hydrolysis by POMs. The strong denaturing effect caused by SDS results in acceleration of hydrolysis by exposing the cleavage sites to the POMs. CHAPS does not cause structural changes in Cyt c and therefore, similar interactions with the POM are expected as in the absence of surfactant, which is reflected in the negligible effect of CHAPS on kinetics of Cyt c hydrolysis.

In order to further understand these phenomena on a molecular level, we investigated the effect of surfactants and POMs on the structure of Cyt c with the help of tryptophan fluorescence spectroscopy.

### Tryptophan fluorescence of Cyt c in the presence of CeK, ZrK, and surfactants

Cyt c is a small protein consisting of 104 amino acids with only one tryptophan residue (Trp) and a covalently bound heme group. In its native state Trp is hydrogen bonded to a propionate of the heme group. The proximity of the heme causes an intrinsic quenching of Trp fluorescence due to the energy transfer from the excited Trp residue to the heme group. When Cyt c unfolds, the distance between Trp and the heme group increases, which enhances the fluorescence intensity (Myer et al., 1980; Elöve et al., 1992; Bychkova et al., 1996; Kamatari et al., 1996; Konermann and Douglas, 1997; Rodriguez-Cruz et al., 2001; Tsong, 2002). Figure S5 shows the effect of the surfactants on the Trp fluorescence of Cyt c.

**Figure 7:**
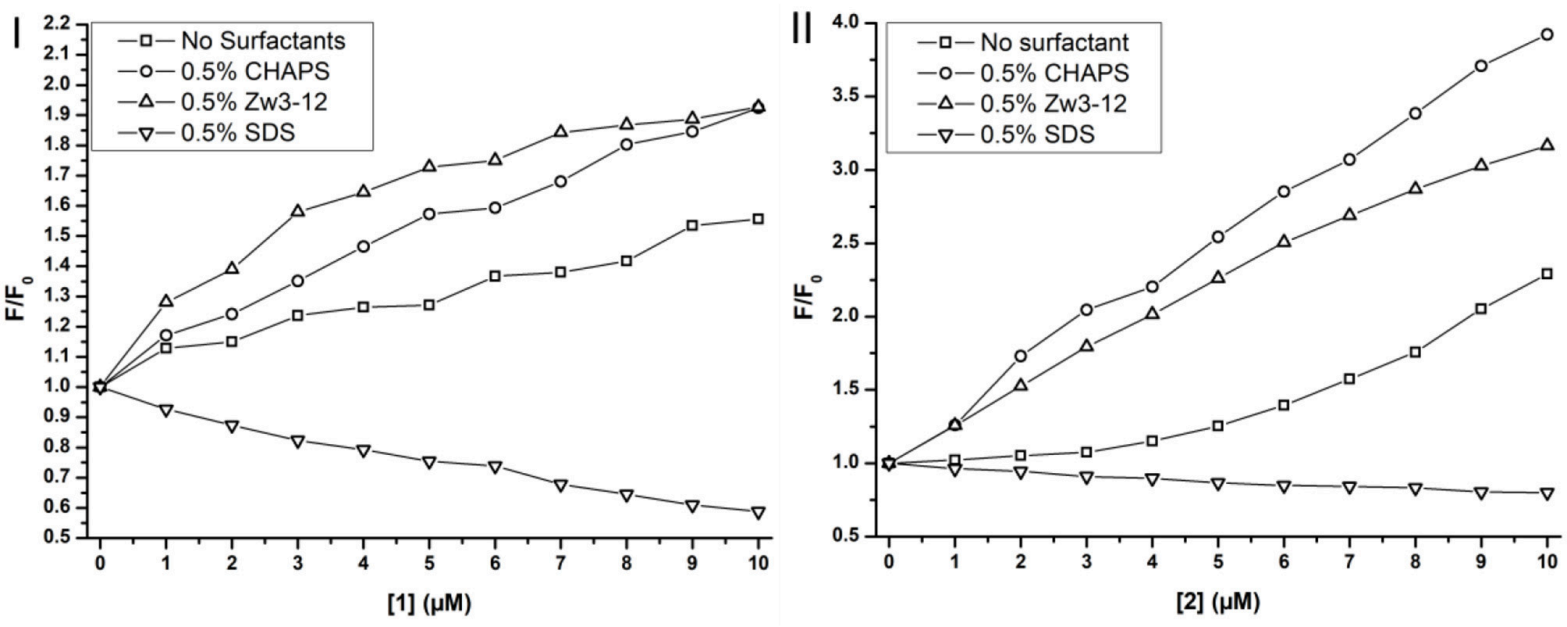
Trp fluorescence of Cyt c in function of the concentration of CeK [1] **(I)** and ZrK [2] **(II)** in μM in the presence or absence of surfactants. The fluorescence is expressed as the ratio of the fluorescence, F, over the fluorescence at [1] or [2] = 0 mM, F_0_.

Previously, Soret CD spectroscopy indicated that CHAPS and Zw3-12 did not alter the tertiary structure of Cyt c, while SDS caused severe structural changes of the protein (see Figure S5). These observations were confirmed with Trp fluorescence spectroscopy. In the presence of 0.5% Zw3-12 or 0.5% CHAPS the intensity of fluorescence is increased by factor of 3, which implies that these surfactants do not cause severe unfolding of Cyt c. SDS, however, amplified the fluorescence to nearly 62 times at even lower concentrations (0.2%). Increasing the SDS concentration did not result in further increase of fluorescence, indicating that Cyt c is already completely unfolded in the presence of 0.2% SDS. The effect of adding **CeK** and **ZrK** POMs to Cytc:surfactant mixtures is shown in Figure 7.

Adding metal substituted POMs to Cyt c in absence of any surfactants causes a slight increase in fluorescence intensity. The intensity increased approximately 1.5 and 2.2 times upon adding 10 μM of **CeK** and **ZrK**, respectively. This indicates that Cyt c partially unfolds upon binding to these POMs, but the effect of **ZrK** is slightly more pronounced than the effect of **CeK**. A partial unfolding of Cyt c was already observed in the presence of Zw3-12 or CHAPS, but adding 10 μM of **CeK** resulted in doubling of the emission intensity in the presence of 0.5% CHAPS or 0.5% Zw3-12. **ZrK** increases the Cyt c fluorescence intensity approximately 3 and 4 times at 10 μM in the presence of 0.5% CHAPS and Zw3-12, respectively. Both CD and Trp fluorescence spectroscopy demonstrated that SDS completely unfolds Cyt c. Adding **CeK** or **ZrK** to a Cyt c/SDS solution quenched the fluorescence. This quenching appeared to be static in nature and could be fitted to a derived Stern-Volmer equation (see Figure S6) (Lakowicz, 2007). The binding constants were determined to be (3 ± 1) × 10^4^ M^−1^ and (4 ± 1) × 10^3^ M^−1^ for **CeK** and **ZrK**, respectively. The order of magnitude difference might be explained by a different counterion present in these POMs. While **CeK** contains potassium cations, **ZrK** has diethylammonium (Et_2_${\text{NH}}_{2}^{+}$). Potassium might compensate the negative charge of SDS by making it appear less anionic to **CeK**. Et_2_${\text{NH}}_{2}^{+}$ however has a positive charge that is partly shielded by the ethyl groups, making it less capable of shielding the negative charge of SDS. Because of this inefficient shielding, SDS will cause a stronger repulsion of the polyanionic POM **ZrK** than it does to POM **CeK**. However, all Trp fluorescence measurements indicate that both **CeK** and **ZrK** bind to Cyt c in the presence or in the absence of surfactants.

### Speciation of CeK in the presence of surfactants

**Figure 8:**
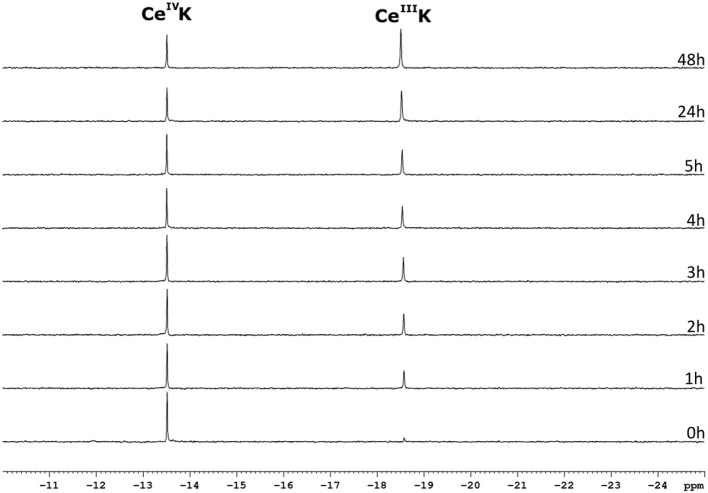
^31^P NMR spectra of a solution of 3.23 mM CeK, 3.32 μM of Cyt c and 0.5% CHAPS in a 10 mM sodium phosphate buffer at pH 7.4. The sample was incubated at 60°C and a ^31^P NMR spectrum was recorded at the time intervals stated to the right of the spectrum. The peaks at −13.5 ppm and −18.6 ppm correspond to the Ce^IV^K and Ce^III^K species, respectively.

The Ce^IV^ substituted Keggin POM, **CeK**, is known to undergo reducion during incubation with proteins (Stroobants et al., 2013; Sap et al., 2016). Luckily, the reduction of the POM does not result in oxidative cleavage of peptide bonds (Stroobants et al., 2013; Sap et al., 2016), as the side chains of amino acids that contain aromatic residues (Trp, Tyr, or Thr), thiols (Cys) and thioethers (Met) are the most likely targets of the oxidation by Ce^IV^. The resulting Ce^III^ substituted POM is thought to be less active as a protease due to the loss in Lewis acidity. The Ce^IV^ → Ce^III^ reduction can be monitored with ^31^P NMR spectroscopy as both species have a distinctly different chemical shift. The Ce^IV^ substituted Keggin is characterized by a ^31^P resonance at ca.−13.5 ppm, while the Ce^III^ species shows a peak at−18.6 ppm. Monitoring the reduction of **CeK** in the presence of the three different surfactants might explain the differences in hydrolysis yields observed above. Figure 8 shows the progress of the reduction of **CeK** in the presence of Cyt c and 0.5% CHAPS over the course of 48 h.

**Figure 9:**
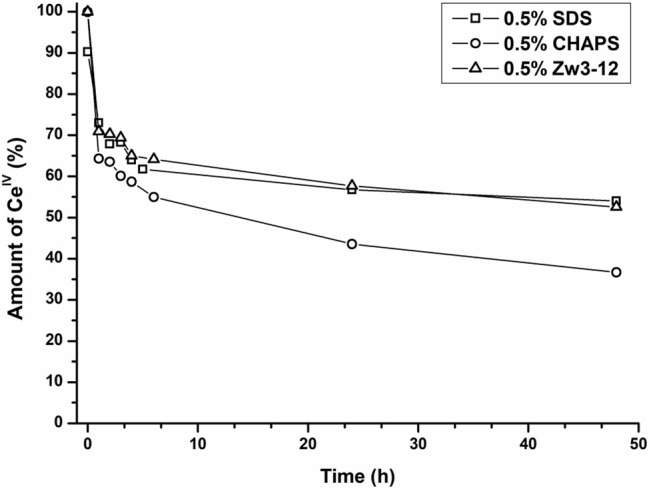
The relative amount of Ce^IV^K in function of time in the presence of Cyt c and 0.5% SDS (square), 0.5% CHAPS (circle) or 0.5% Zw3-12 (triangle).

As can be seen from Figure 8, the amount of the Ce^IV^ POM species decreases over time in favor of the Ce^III^ POM. The relative amount of Ce^IV^ after incubation at 60°C in the presence of different surfactants is plotted as function of time in Figure 9.

In the presence of 0.5% CHAPS, the reduction of Ce^IV^ is accelerated significantly in comparison to SDS and Zw3-12. After 48 h in the presence of 0.5% CHAPS only 38% of Ce^IV^ POM was present, while 54 and 53% of Ce^IV^ POM was observed in the presence of 0.5% SDS and 0.5% Zw3-12, respectively. This decrease of the active catalyst in the presence of CHAPS might explain the slightly lower hydrolysis yield in the presence of this surfactant. However, the strong inhibition of Zw3-12 on hydrolysis appears to originate from its effect on the Cyt c structure in solution and not on its impact on the reduction of Ce^IV^ POM catalyst.

## Conclusions

This study has shown that in the presence of SDS, Zw3-12 or CHAPS surfactants, which are commonly used for solubilizing hydrophobic proteins, the specificity of **CeK** or **ZrK** toward hydrolysis of Cyt c does not change. Zw3-12 and CHAPS did not have a large effect on the secondary and tertiary structure of Cyt c, and therefore the same selectivity as in the absence of the surfactants was observed. In the presence of SDS unfolding of Cyt c secondary and tertiary structure were observed, indicating a large degree of a denaturation. Similar structural changes were observed in the presence of a 50 μM concentration of **CeK** or **ZrK**, indicating that POM binding to Cyt c results in large protein unfolding, both in the absence or presence of all three surfactants. This finding might explain why surfactants have no noticeable effect on the selectivity of **CeK** and **ZrK**. Recent work has reported on the specificity of [Zr(α_2_-P_2_W_17_O_61_)_2_]^16−^ toward HSA in the absence or presence of CHAPS (Sap et al., 2017), in which 7 distinct cleavage sites were observed in the presence of 0.5% CHAPS whereas 4 were determined in the absence of any surfactant. This was explained by the fact that the partial unfolding of HSA in the presence of CHAPS allowed the POM access to peptide bonds, which were previously buried in the native structure of the protein. This different effect of the surfactants on the hydrolysis of HSA and Cyt c is probably due to different size of these proteins. While HSA is a large protein of 66.4 kDa (609 amino acids), Cyt c is a relatively small protein consisting of 12.4 kDa (104 amino acids). In their native state, larger proteins can accommodate POMs without causing drastic structural changes (Goovaerts et al., 2013, 2015; Stroobants et al., 2014a), whereas the smaller proteins undergo significant structural changes when binding with POMs (Stroobants et al., 2013). This work shows that the structural changes in Cyt c caused by surfactants are similar to those caused by POMs, hence the same specificity in the absence or presence of surfactants was observed.

The hydrolysis of Cyt c by **CeK** was increased in the presence of SDS, but decreased in the presence of CHAPS, and was nearly inhibited in the presence of Zw3-12. The tertiary structure of Cyt c in the presence of SDS closely resembles the structure in the presence of 50 μM **CeK**, but the structure in the presence of CHAPS and Zw3-12 is closely related to the native structure. These findings indicate that for Cyt c hydrolysis to occur, large unfolding of the protein is needed in order to accommodate the POMs. While SDS readily unfolds Cyt c, the protein remains largely folded in the presence of CHAPS and Zw3-12. Addition of POMs to Cyt c solutions in CHAPS results in unfolding of the structure allowing the interaction with POMs to occur and results in protein hydrolysis. Zw3-12, however, locks Cyt c in a conformation that resists unfolding upon addition of POM, and therefore results in nearly complete inhibition of protein hydrolysis.

These findings, which reveal the effect of different surfactants on the reactivity of POMs, may be an important step forward in developing metal-substituted POMs as a potential class of metalloproteases for the hydrolysis of hydrophobic and membrane proteins.

## Experimental

### Materials

Deuteriumoxide (D_2_O), N,N,N′,N′-tetramethylethylenediamine (TEMED), ammonium persulphate (APS), phosphotungstic acid hydrate (H_3_[PW_12_O_40_]·xH_2_O), glycine, disodium phosphate (Na_2_HPO_4_), sodiu, dodecyl sulfate (SDS), horse heart cytochrome c (Cyt c) and bromophenol blue were bought from Sigma-Aldrich. Zirconium oxychloride octahydrate and diethyl ether were purchased from ChemLab. Aqueous hydrochloric acid (37%), potassium hydrogen carbonate and ammonium cerium^IV^ nitrate (CAN) were obtained from Acros organics. Ethanol, sodium hydrogen carbonate, aqueous *ortho*-phosphoric acid (85%), diethyalaminehydrochloride and protein ladders were acquired from Thermo Fisher Scientific. Methanol and monosodiumphosphate (NaH_2_PO_4_) were purchased from VWR. Potasium chloride, tris(hydroxymethyl)aminomethane (TRIS) and acrylamide:bisacrylamide (29:1) solution (30%) were procured from Applichem. [Ce(α-PW_11_O_39_)_2_]^10−^ (**CeK**) was synthesized according to (Griffith et al., 2000) [Zr(α-PW_11_O_39_)_2_]^10−^ (**ZrK**) was synthesized following a slightly altered procedure from (Sokolov et al., 2007).

### Methods

#### Circular dichroism spectroscopy

Solution containing 10 μM Cyt c neat or in the presence of 0.5% SDS or 0.5% Zw3-12 were prepared in a 10 mM sodium phosphate buffer at pH 7.4. The concentration of **CeK** and **ZrK** was increased incremental from 0 to 50 μM in 10 μM steps. The samples were kept at a constant temperature of 25 ± 0.1°C during the recording of all spectra. The CD spectra were recorded with a JASCO-1500 spectrometer directly after the samples were prepared. The far-UV (180–260 nm) and Soret (300–500 nm) CD spectra were recorded with a 1 mm and 10 mm quartz cuvette, respectively. The resulting spectra are averaged over 3 accumulations with bandwidth of 1 nm. All spectra were corrected for background effects by substracting the spectra of the respective buffer solutions. The machine response (θ in mdeg) was converted to mean residue ellipticity ([θ]_MRE_) according to Equation (1): (Wallace and Janes, 2009).

(1) $$\begin{array}{l} {\left[\theta \right]}_{MRE}=\frac{\theta \;\times \;0.1\;\times \;\langle M\rangle }{c_{g}\;\times \;l_{cm}}=\frac{\theta }{c_{M}\;\times \;n\;\times \;l_{mm}} \end{array}$$

Where <*M*> is the mean residue molar weight (equal to Mw/(*n*−1)); *c*_*g*_ and *c*_*M*_ the protein concentration in g/L and mol/L, respectively; *l*_*cm*_ and *l*_*mm*_ the optical path length in cm and mm, respectively and *n* the number of peptide bonds in the protein (equal to the total number of amino acids minus one).

The secondary structural content was calculated using the web service DICHROWEB (Whitmore and Wallace, 2004, 2008) with the CDSSTR algorithm and protein reference set 3 (optimized for the spectral region from 185 to 240 nm) (Sreerama and Woody, 2000).

#### Fluorescence spectroscopy

Samples containing 10 μM Cyt c neat or in the presence of 0.5% SDS, CHAPS or Zw3-12 were prepared in a 10 mM sodium phosphate buffer at pH 7.4. The concentration of **CeK** and **ZrK** was increased from 0 to 10 in 1 μM steps. The samples were kept at ambient temperature during the recording of the spectra. A 10.0 mm quartz cuvette was used to record emission spectra using an Edinburgh Instruments FLS-980 spectrometer. The samples were excited at 295 nm and emission was observed from 300 to 450 nm with a maximum at approximately 333 nm.

#### Hydrolysis experiments

Solutions containing 30 μM Cyt c and 50 equivalents of **CeK** or **ZrK** in the presence or absence of the different surfactants (0.5% CHAPS, SDS or Zw3-12) were prepared in 10 mM sodium phosphate buffer at pH 7.4. The samples were incubated for several days at 60°C and aliquots were taken at several time intervals. SDS-PAGE was used to monitor the progress of the hydrolysis (6% stacking gels and 18% running in 0.1 M Tris-Tricine, 0.1% SDS running buffer).

Samples were mixed in a 2 to 1 ratio with a 3x sample buffer (250 mM DTT, 50% glycerol, 5% SDS, 0.05% bromophenol blue, 225 mM Tris-HCl buffer, pH 6.8) and incubated at 95°C for 5 min. After 3 min of centrifuging, 10 μM of the final sample solution was loaded on an 18% gel. The PageRuler™ Unstained Low Range Protein Ladder of Thermo scientific was used as Mw reference. The gels were run at 300 V and 30 mA/gel current using an OmniPage electrophoretic cell combined with an EV243 power supply. The gels were developed with Coomassie blue or silver staining and analyzed using a GelDoc EZ set-up with the Image Lab software (Bio-Rad, Hercules, CA).

#### ^31^P NMR spectroscopy

Samples containing 32.2 μM Cyt c and 3.23 mM **CeK** neat or in the presence of 0.5% CHAPS, Zw3-12 or SDS were prepared in a 10 mM sodium phosphate buffet at pH 7.4. The samples contained 10% D_2_O and were incubated at 60°C and measured at different time points. The spectra were recorded on a 400 MHz Avance III^+^ spectrometer (Bruker) with a sweep with of 100 ppm, 256 scans, relaxation delay of 2.5 s and the center of the spectrum at 0 ppm. A 25% H_3_PO_4_ solution was used as an external reference to calibrate all spectra.

## Author contributions

All experimental work and data analysis was performed by TQ and TD under guidance of TQ. The manuscript was written by TQ with valuable contributions and corrections of TP-V and PS.

### Conflict of interest statement

The authors declare that the research was conducted in the absence of any commercial or financial relationships that could be construed as a potential conflict of interest.
