# Supplementary material for: Selectivity and Reactivity of ZrIV and CeIV Substituted Keggin Type Polyoxometalates Toward Cytochrome c in Surfactant Solutions
Source: Front Chem. 2018 Aug 28;6:372. doi: 10.3389/fchem.2018.00372 (PMC6121075; doi:10.3389/fchem.2018.00372)
Supplement: Supplementary file 1 [file Presentation_1.PDF]

Supplementary Material

Selectivity and reactivity of Zr^{IV} and Ce^{IV} substituted Keggin type polyoxometalates towards cytochrome c in surfactants solutions

Thomas Quanten, Tessa De Mayaer, Pavletta Shestakova and Tatjana N. Parac-Vogt*

*** Correspondence:** tatjana.vogt@kuleuven.be

1 Supplementary Figures

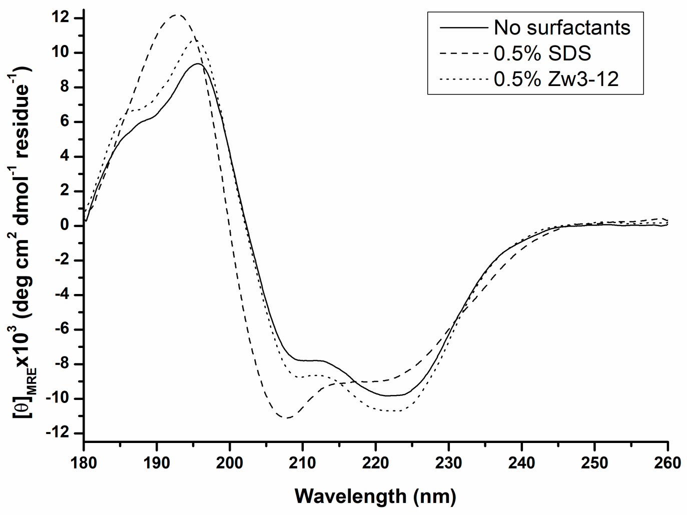

Supplementary Figure 1. The mean residue ellipticity ($[\theta]_{MRE}$) in function of the wavelength of the far-UV CD spectra of 10 µM Cyt c (solid line) and 10 µMCyt c in presence of 0.5% SDS (dashed line) and 0.5% Zw3-12 (dotted line) at 25 °C. All samples were buffered at pH 7.4 with a 10 mM sodium phosphate buffer.

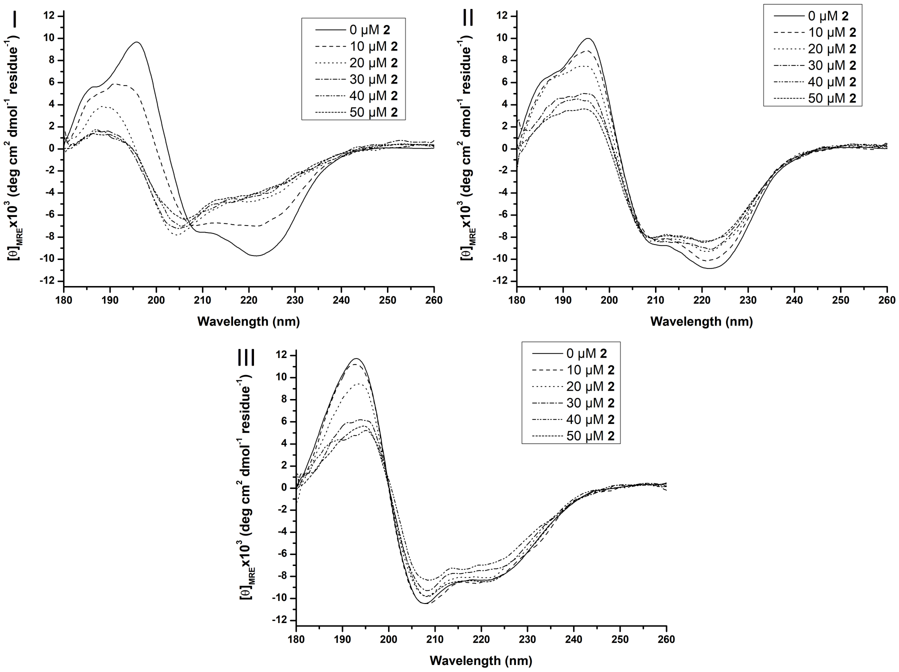

Supplementary Figure 2. CD spectra of 10 μM Cyt c solutions in the presence of increasing concentrations of **ZrK** (**2**) (from 0-50 μM) in absence of surfactants (I), in presence of 0.5% Zw3-12 (II) and 0.5% SDS (III). All samples were buffered at pH 7.4 by a 10 mM sodium phosphate buffer and kept at 25±0.1 °C during measurements.

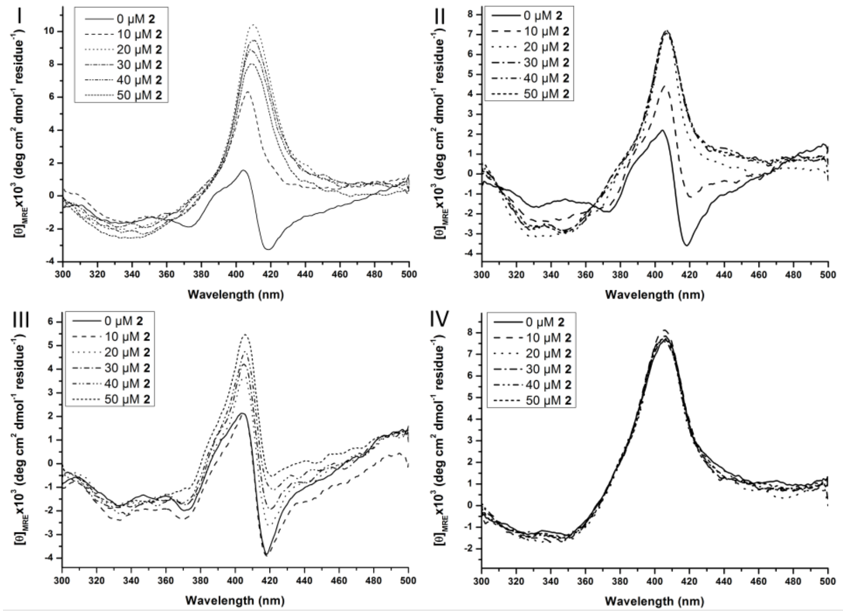

Supplementary Figure 3. CD spectra of 10 μM Cyt c solutions in presence of increasing concentration of **ZrK** (**2**) (from 0-50 μM) in absence of surfactants (I), in presence of 0.5% CHAPS (II), 0.5% Zw3-12 (III) and 0.5% SDS (IV). All samples were buffered at pH 7.4 by a 10 mM sodium phosphate buffer and kept at 25±0.1 °C during measurements.

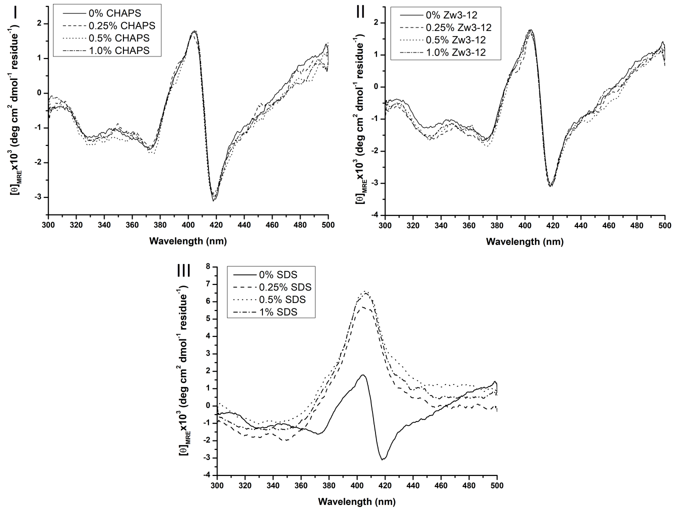

Supplementary Figure 4. Soret region CD spectra of 10 μM Cyt c in presence of increasing concentrations of different surfactants: I. CHAPS, II. Zw3-12 and III. SDS. All samples were buffered at pH 7.4 by a 10 mM sodium phosphate buffer and kept at 25±0.1 °C during measurements.

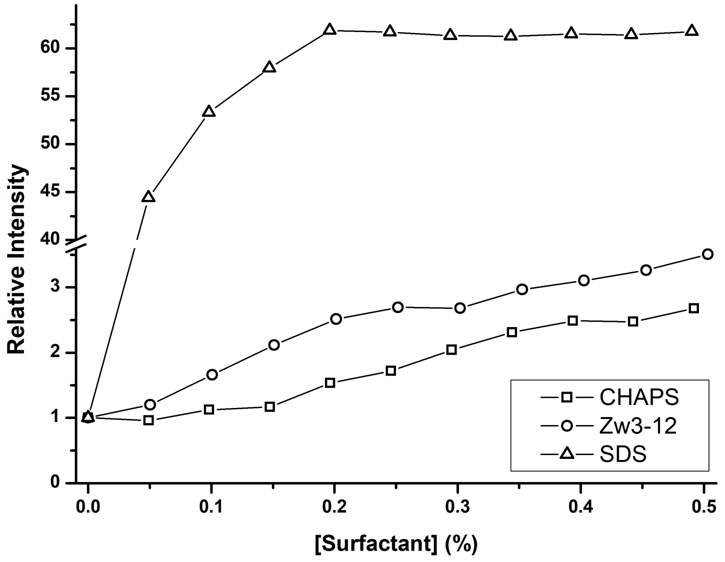

Supplementary Figure 5. The relative intensity of the Trp fluorescence plotted in function of the surfactant concentration. The relative intensity equals the intensity divided by the intensity at 0 mM surfactant. All samples were buffered at pH 7.4 (10 mM sodium phosphate)

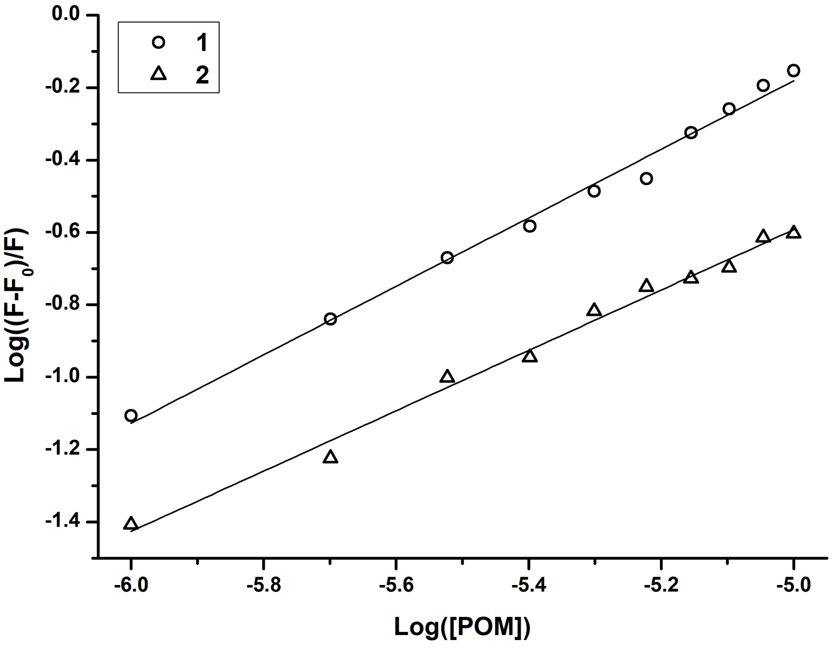

Supplementary Figure 6. The plotted derived Stern-Volmer of the fluorescence of Cyt c quenched by **CeK** (**1**) or **ZrK** (**2**) in the presence of 0.5% SDS. The lines are the fitted curves according to **Eq. S1.** The binding constants that were calculated to be $(3\pm1)\times10^{4}$ M^{-1} for **CeK** and $(4\pm1)\times10^{3}$ M^{-1} for **ZrK** and the complex stoichiometry was found to be 0.8:1 and 1:1 (POM:Cyt c) for **CeK** and **ZrK**, respectively.

1.1 Supplementary tables

Supporting Table 1. The ST content of Cyt c in the absence or presence of 0.5% SDS or Zw3-12 as determined by the CDSSTR algorithm (protein reference set 3).

Surf.	α (%)	β (%)	T (%)	U (%)
-	32	11	23	33
0.5% Zw3-12	35	8	23	34
0.5% SDS	34	17	23	25

Surf., surfactant; α, α-helix; β, β-strand; T, turns; and U, unordered

Supporting Table 2. The ST content of Cyt c in the absence (**bold** values between brackets) or presence (values outside brackets) of 50 µM POM in the absence or presence of 0.5% SDS or Zw3-12 as determined by the CDSSTR algorithm (protein reference set 3).

Surf.	POM (50 µM)	α (%)	β (%)	T (%)	U (%)
None	**CeK**	8 (**32**)	34 (**11**)	24 (**23**)	34 (**33**)
	**ZrK**	8 (**32**)	36 (**12**)	23 (**23**)	34 (**31**)
0.5% Zw3-12	**CeK**	19 (**35**)	27 (**8**)	23 (**23**)	31 (**34**)
	**ZrK**	21 (**35**)	25 (**8**)	23 (**23**)	31 (**34**)
0.5% SDS	**CeK**	30 (**34**)	17 (**17**)	24 (**23**)	29 (**25**)
	**ZrK**	31 (**33**)	17 (**18**)	23 (**22**)	28 (**26**)

Surf., surfactant; α, α-helix; β, β-strand; T, turns; and U, unordered. The values inside and outside of the brackets are the ST contents in the absence or presence of POMs, respectively.

1.2 Equations

$$Log\left(\frac{F_0-F}{F}\right) = Log(K_q) + n \times Log([Q]) \qquad \textbf{Eq. S1}$$

The derived Stern-Volmer equation where F_0 is the unquenched fluorescence intensity, F represents the quenched fluorescence intensity in the presence of Q, K_q is the quenching constant, n the number of bound quencher molecules and $[Q]$ signifies the concentration of the quencher Q.
